# Scaling Structure Aware Virtual Screening to Billions of Molecules with SPRINT

**Published:** 2025-01-20

**Authors:** Andrew T. McNutt, Abhinav K. Adduri, Caleb N. Ellington, Monica T. Dayao, Eric P. Xing, Hosein Mohimani, David R. Koes

**Affiliations:** 1 Computational and Systems Biology, University of Pittsburgh, Pittsburgh, PA, USA.; 2 Computational Biology, Carnegie Mellon University, Pittsburgh, PA, USA.; 3 Mohamed Bin Zayed University of Artificial Intelligence, Masdar City, Abu Dhabi.; 4 Petuum Inc., Pittsburgh, PA.

**Keywords:** virtual screening, structure-based drug discovery, protein language models

## Abstract

Virtual screening of small molecules against protein targets can accelerate drug discovery and development by predicting drug-target interactions (DTIs). However, structure-based methods like molecular docking are too slow to allow for broad proteome-scale screens, limiting their application in screening for off-target effects or new molecular mechanisms. Recently, vector-based methods using protein language models (PLMs) have emerged as a complementary approach that bypasses explicit 3D structure modeling. Here, we develop SPRINT, a vector-based approach for screening entire chemical libraries against whole proteomes for DTIs and novel mechanisms of action. SPRINT improves on prior work by using a self-attention based architecture and structure-aware PLMs to learn a co-embedding space for drugs and targets, enabling efficient binder prediction, search, and retrieval. SPRINT achieves SOTA enrichment factors in virtual screening on LIT-PCBA, DTI classification benchmarks, and binding affinity prediction benchmarks, while providing interpretability in the form of residue-level attention maps. In addition to being both accurate and interpretable, SPRINT is ultra-fast: querying the whole human proteome against the ENAMINE Real Database (6.7B drugs) for the 100 most likely binders per protein takes 16 minutes. SPRINT promises to enable virtual screening at an unprecedented scale, opening up new opportunities for *in silico* drug repurposing and development. SPRINT is available on the web as *ColabScreen*: https://bit.ly/colab-screen.

## 1 Introduction

Virtual screening has emerged as a powerful tool for predicting drug-target interactions (DTIs) and guiding experimental efforts, but conventional structure-based methods like molecular docking are often too slow for proteome-scale analyses [1]. This limitation hinders their application in crucial parts of the drug discovery process such as off-target prediction [2]. The need for scalable and interpretable virtual screening methods is particularly evident in, for example, antimicrobial drug discovery. The rapid emergence of antimicrobial-resistant pathogens poses a severe threat to public health [3], necessitating the development of new antibiotics with novel mechanisms of action to combat cross-resistances [4]. Effective antimicrobial virtual screening requires methods that are not only fast and scalable but also interpretable, enabling researchers to: 1) identify new drug candidates with on-target effects across thousands of microbial proteomes and minimal off-target effects in humans and 2) provide interpretations for predicted DTIs and potential mechanisms of action.

Recently, vector-based virtual screening has been proposed as an alternative to structure-based screening to efficiently predict DTIs, leveraging vector featurizations for molecules [5] and sequence models for protein targets [6–8]. One method, ConPLex [2], proposes co-embedding molecules and proteins into a shared vector space, where the distance between entities is proportional to interaction likelihood. This effectively reduces the task of computing a DTI to a dot product in the co-embedding space, enabling the screening of millions of molecules against the entire human proteome in 24 hours. However, ConPLex does not scale favorably when identifying DTIs across thousands of bacterial and fungal proteomes, and it cannot provide explanations of its DTI predictions. Similarly, DrugCLIP [9, 10] aligns the embeddings of protein pocket structures and ligands with contrastive learning such that similarity encodes the probability of interaction. They demonstrate state-of-the-art (SOTA) results on virtual screening benchmarks with a fraction of the compute time needed for other structure-based virtual screening methods. Their approach is restricted to structures for which binding pockets can be predicted using pocket detection algorithms or homology-based approaches; however, [11] estimated that almost half of all structured domains may lack obvious pockets in their experimental structures.

In this work, we propose SPRINT (Structure-aware PRotein ligand INTeraction) for fast and accurate vector-based DTI predictions. SPRINT implicitly uses structure information by featurizing proteins with SaProt [8], a transformer model trained by augmenting the standard amino acid vocabulary with discrete structure-tokens [12]. Instead of averaging the per-residue embeddings from SaProt, SPRINT uses a multi-head attention pooling scheme to learn a sequence-dependent aggregation for protein representation.

SPRINT is extremely fast: querying a single protein target against the ENAMINE REAL (6.7B drugs) database and predicting its top-100 binders takes 7ms when utilizing ChromaDB[13]. Our main contributions are summarized as:

- Achieving excellent performance on DTI classification (Table 1), virtual screening (Table 2), and binding affinity prediction (Table 3).
- Enhance the second place method in the second critical assessment of computational hit-finding experiments (CACHE) challenge.
- Enabling pan-proteome-scale DTI screens using vector store and retrieval, scaling to billions of molecules.
- Improving molecular property prediction using the molecule co-embeddings learned via predicting DTIs.
- Investigating attention weights and visualizing attention maps to interpret model predictions.

Our software is available on our GitHub repository: https://github.com/abhinadduri/panspecies-dti and is also available on the web as *ColabScreen*: https://bit.ly/colab-screen.

## 2 Results

### 2.1 Multi-head attention pooling improves DTI prediction

A limitation of the ConPLex framework is that it averages the per-residue embeddings obtained from PLMs [2]. As much of the relevant signal for DTIs is located in the binding pocket residues, average pooling can noise the contact map information carefully learned by PLMs through self-attention [14], particularly in the longer sequence-length regime. Retraining the ConPLex model with an attention-based, learned aggregation function [15] achieves SOTA predictive scores for DTIs on most benchmarks (Table 1), even when using the same ProtBert model [6].

To see how the learned aggregation scales with the available training data, we trained a SPRINT model on a huge dataset of DTIs, which we refer to as “MERGED”, [16] combining DTI data from PubChem [17], BindingDB [18], and ChEMBL [19] (further details can be found in Appendix C). Our largest model, SPRINT, uses 3-layer MLPs to encode molecules and proteins after multi-head attention pooling, in contrast to SPRINT-sm’s single-layer MLPs. SPRINT exhibits overfitting on the BIOSNAP, BindingDB, and DAVIS datasets but significantly improves performance on the much larger MERGED dataset (Table 1), confirming that there is value in scaling the SPRINT model size as we increase the amount of training data.

LIT-PCBA[26] is a challenging, commonly used virtual screening benchmark that addresses biases in the previously used DUD-E dataset [27] to explicitly enable validation of machine learning models. However, the activity labels in LIT-PCBA are derived from dose-response bioassays reported in PubChem [17], which may introduce noise and variability that could potentially impact the reliability of model evaluations. To evaluate the performance of SPRINT models at virtual screening on LIT-PCBA in the zero-shot setting, we pre-trained the deeper SPRINT (16M) model on the MERGED dataset after removing all protein sequences with ≥ 90% sequence homology to the LIT-PCBA set using MMSeqs2 [28]. We see that the structure-aware SPRINT models significantly outperform competitor methods in AUROC, BEDROC (alpha = 0.85), and across all enrichment factor thresholds (Table 2). The SPRINT models outperform similarly sized models trained using ProtBert featurizations and multi-head attention pooling (SPRINT-ProtBert), and models trained using SaProt featurizations and average pooling (SPRINT-Average) demonstrating the importance of structure and self-attention.

We further evaluate SPRINT for binding affinity prediction on the Therapeutic Data Commons (TDC) ‘BindingDB_Patent’ Leaderboard[29]. We see in Table 3 that SPRINT using both ProtBert, ESM2, and SaProt PLMs are competitive with the top-ranking model on the leaderboard, Otter-Knowledge-Ensemble[30]. This is notable, as the top ranking model is an ensemble of four knowledge graph-refined protein and ligand representations with each set of representations fine-tuned on a separate dataset of protein-ligand interactions. All of the models start with ESM2 and Morgan fingerprint embeddings for the protein and ligand, respectively. We see that the SPRINT-ESM2 model performs similar to the Otter-Knowledge-Ensemble, an ensemble of refined ESM2 and Morgan representations. The SPRINT models match the performance while only observing the BindingDB_Patent training set. Interestingly, both ProtBert and ESM2 outperform SaProt in this task, despite SaProt performing better in previous tasks (Tables 1 and 2).

### 2.2 Enhancing Real-World Virtual Screening in CACHE2

**Fig. 1:**
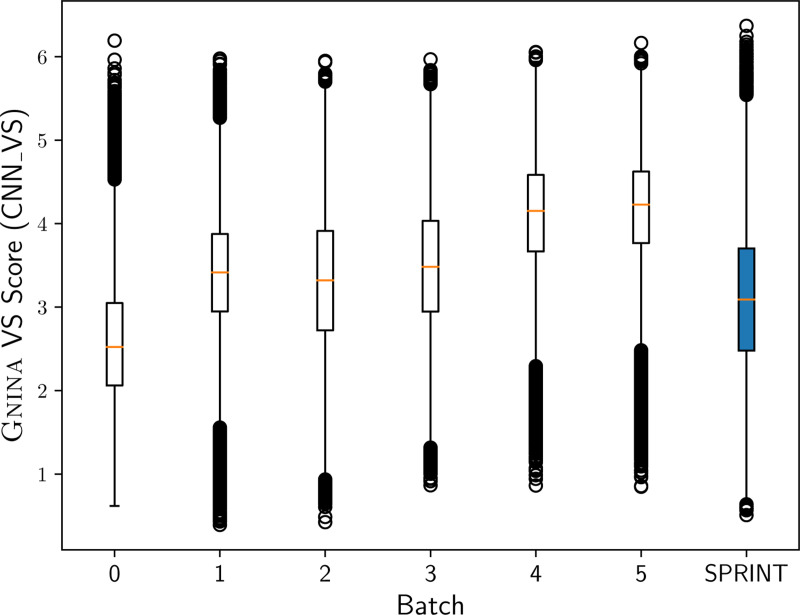
Gnina CNN VS scores of the molecules found during DeepDocking and the molecules picked with SPRINT.

The Critical Assessment of Computational Hit-Finding Experiments (CACHE) [31] serves as a benchmark for prospective evaluation of virtual screening methods through experimental validation of predicted hits. CACHE challenge 2 focused on the RNA binding site of SARS-CoV-2 NSP13, a helicase representing the most conserved site across coronaviruses [32] with no known inhibitors. . Crystal structures with bound fragments in the RNA-binding site exist, with PDB ID 5RLZ used for virtual screening. The second-place team [33] implemented a DeepDocking [34] approach using Gnina [1, 23]. They first filtered the Enamine REAL database for drug-like properties, reducing it to 4.4 billion molecules. Then, a random sample of 100,000 molecules (Batch 0) was docked against the 5RLZ structure using Gnina, and a surrogate model was trained to predict the docking score directly from a fingerprint of the molecule [5]. The entire database was scored with the surrogate model, and the top-scoring 100,000 molecules (Batch 1) were docked again with Gnina to produce training data for a new surrogate model. This process was repeated until the docking scores converged (Batch 5), as shown in Figure 1.

To evaluate the utility of SPRINT in filtering a compound database for high-likelihood binders and speeding up the DeepDocking process, we used SPRINT, pretrained on the MERGED dataset, to predict DTI scores for the filtered Enamine REAL database against the same virtual screening structure (5RLZ). We selected the highest-scoring 100,000 molecules from the filtered database and docked them using Gnina, following the same docking protocol as [33]. The virtual screening scores (CNN VS [1]) of our docked compounds are compared to the batches of Gnina DeepDocking in Figure 1. Molecules selected by SPRINT had a higher average CNN VS score (3.10) than Batch 0 of DeepDocking (2.59), and are more distributionally similar to Batches 1, 2, and 3. Notably, SPRINT finds 16 diverse, high-scoring molecules (CNN VS > 6) shown in Figure D2, while only 6 high-scoring molecules are found through DeepDocking (with one high-scoring molecule found during the initial random selection, Batch 0).

### 2.3 Structure-aware protein embeddings improve attention maps

**Fig. 2:**
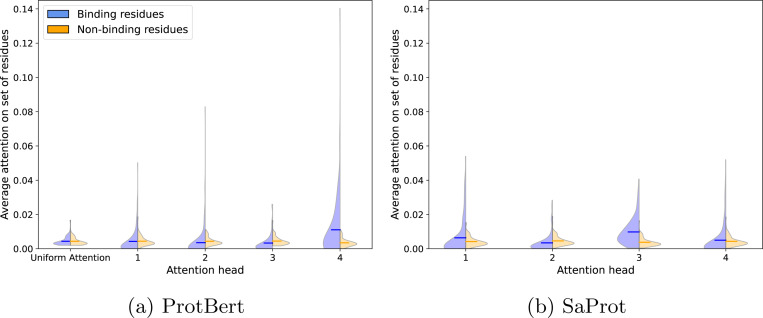
Comparing the average attention weight of binding and non-binding residues on our set of 109 single-chain protein-ligand binding structures after training on the MERGED Dataset (Methodology detailed in Appendix E). We visualize the Protbert and SaProt models trained with equal positive and negative sampling. The horizontal line indicates the average across the proteins. Visualizations of the ProtBert and SaProt models trained with increased negative sampling are in Figure E3).

**Fig. 3:**
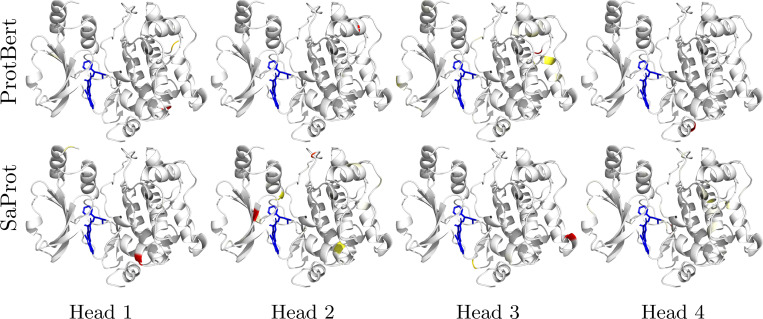
Analyzing the attention on PDB ID 2X4Z using ProtBert and SaProt models trained with equal ratio of positive and negative examples (identical models trained with different initial random seeds visualized in Figures F5 and F7; models trained with increased negative sampling visualized in Figures F4 and F6). Each column is a different attention head. Gradient from white to red indicates the attention weight, where white is no attention and red is max attention for that head. The ligand is shown in blue.

Following training on the MERGED dataset with either ProtBert or SaProt as the PLM backbone for SPRINT, we analyze the attention patterns learned on a set of single-chain protein-ligand binding structures. We find that models with the greatest enrichment factors on LIT-PCBA, which are trained by sampling many negative DTIs for each positive DTI, have sparse attention that focuses on residues very distant from ligand interactions (Figures E3, F4, and F6). Therefore, we focus our attention analysis on SPRINT models trained with equal positive and negative sampling (LIT-PCBA results are provided in Table C2). All but one of the ProtBert attention heads attend less to the binding residues than they do to the non-binding residues (Figure 2). By introducing explicit knowledge of the protein’s structure with SaProt, we find more heads attending to the binding residues than the non-binding residues on average. We visualize the attention on the bound structure of a serine/threonine kinase (PDB ID: 2X4Z) in Figure 3 (additional visualizations provided in Appendix F). Both models have sparse attention maps, with only a handful of residues with non-trivial attention values per head. Attention head 2 of the SaProt model pulls out several residues near the binding site of the kinase, while none of the Protbert heads have much, if any, attention on the residues near the binding site, instead focusing on the edges of the protein.

Although the learned aggregation layer allows for model interpretation, we find there is little biological relevance for the attention patterns of the model at its current scale. For example, we compare the attention patterns to a multiple sequence alignment (MSA) of 497 human kinase domains from [35] and find that both models attend to non-conserved residues of the kinase that could identify the exact protein with a small residue fingerprint.

### 2.4 SPRINT enables querying binding partners from 5132 proteomes

**Fig. 4:**
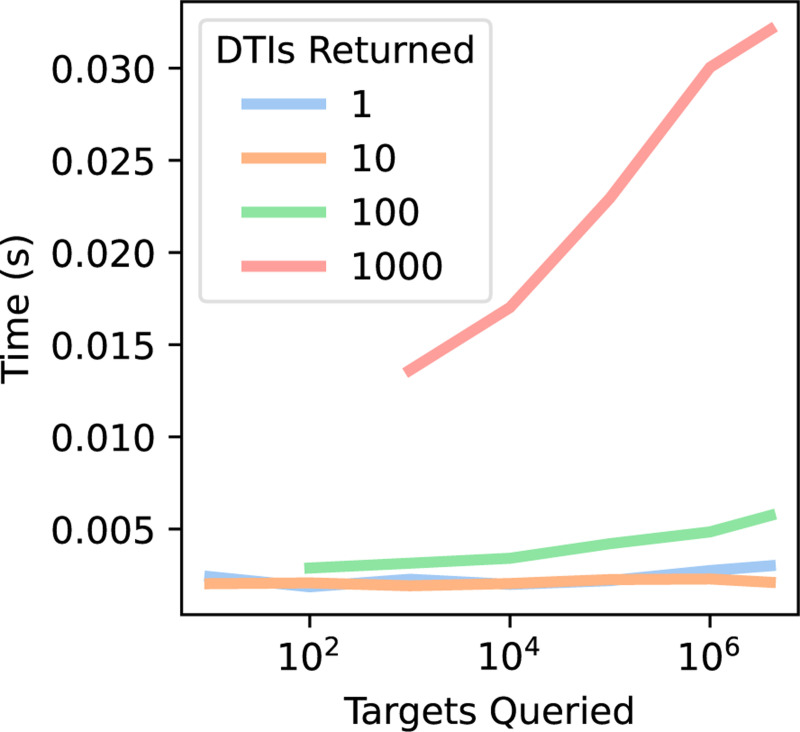
Times for predicting the top DTIs for a ligand using vector search.

To demonstrate the utility of SPRINT at the pan-species-proteome scale, we constructed a dataset of 5,043 bacterial proteomes, 88 fungal proteomes, and the human proteome, containing 4,291,525 total protein sequences. To store and query the co-embeddings, we use the Chroma vector store[13], a tool developed for semantic search and retrieval-augmented generation in natural language processing. The scaling properties of this framework are highly favorable (Fig. 4): querying a ligand for the 100 most likely binders against the entirety of UniProt (60M sequences) takes 0.0001s, and querying all 2e6 molecules in CHEMBL for each of their 10 most likely binders against the 4.3M proteins in our multi-species dataset takes less than 4 hours. As a proof of concept, we co-visualized several antimicrobials and drugs with their known protein targets across microbial proteomes (Fig. A1).

### 2.5 Pre-training to predict DTIs improves property prediction

To benchmark the usefulness of DTI co-embeddings for marginal property prediction, e.g., predicting the properties of a compound only from its molecular graph, we computed SPRINT DTI co-embeddings for several drug-like compounds [36] and natural products [37]. Concatenating the SPRINT-sm molecule co-embedding to a Morgan fingerprint consistently outperformed an equivalently sized neural network using only the Morgan fingerprint as input (Table B1). However, we observe that using only the SPRINT embedding in these tasks degrades performance, suggesting that SPRINT embeddings can synergistically enhance traditional fingerprints.

## 3 Methods

To enable fast and accurate screens, we seek a co-embedded representation of drugs and protein targets where a simple similarity metric indicates binding likelihood. Let D and T denote the random variables representing drugs and targets, f and g denote the choice of frozen drug and target encoders, and $C_{d}$ and $C_{t}$ denote modality-specific neural networks that project drug and target embeddings, respectively, into a shared co-embedding space. Let Y denote the random variable representing drug-target interaction, where Y=1 denotes an interacting pair, and Y=0 denotes a non-interacting pair. Denoting latent co-embeddings $Z_{d}=C_{d}(f(D))$ and $Z_{t}=C_{t}(g(T))$, our model is:

(1) $$P\left(Y=1|Z_{d},Z_{t}\right)=\sigma \left(\alpha \frac{Z_{d}}{\left\|Z_{d}\right\|}\cdot \frac{Z_{t}}{\left\|Z_{t}\right\|}\right)$$

where σ denotes the sigmoid activation function, and α is a constant scaling factor chosen to saturate the range of the sigmoid function, as unscaled cosine similarity ranges from (−1,1). In our implementation, we choose α=5. Our goal through training is to learn $C_{d}$ and $C_{t}$ that minimize binary cross-entropy loss against ground truth binding and non-binding pairs.

In addition to classifying drug-target binding, SPRINT is capable of predicting binding affinity without changing learned portions of the model. By replacing the cosine similarity with a dot-product and removing the final sigmoid, we can leverage SPRINT as a binding affinity predictor (Equation 2):

(2) $$p\hat{K}=\left(Z_{d}\cdot Z_{t}\right)$$

where $p\hat{K}$ is the predicted $pK=-log_{10}\left(K_{d}\right)$ to simplify training of the model on affinity values. Binding affinity regression models are trained to minimize mean square error loss against ground truth binding affinity by updating the weights of $C_{d}$ and $C_{t}$.

**Fig. 5:**
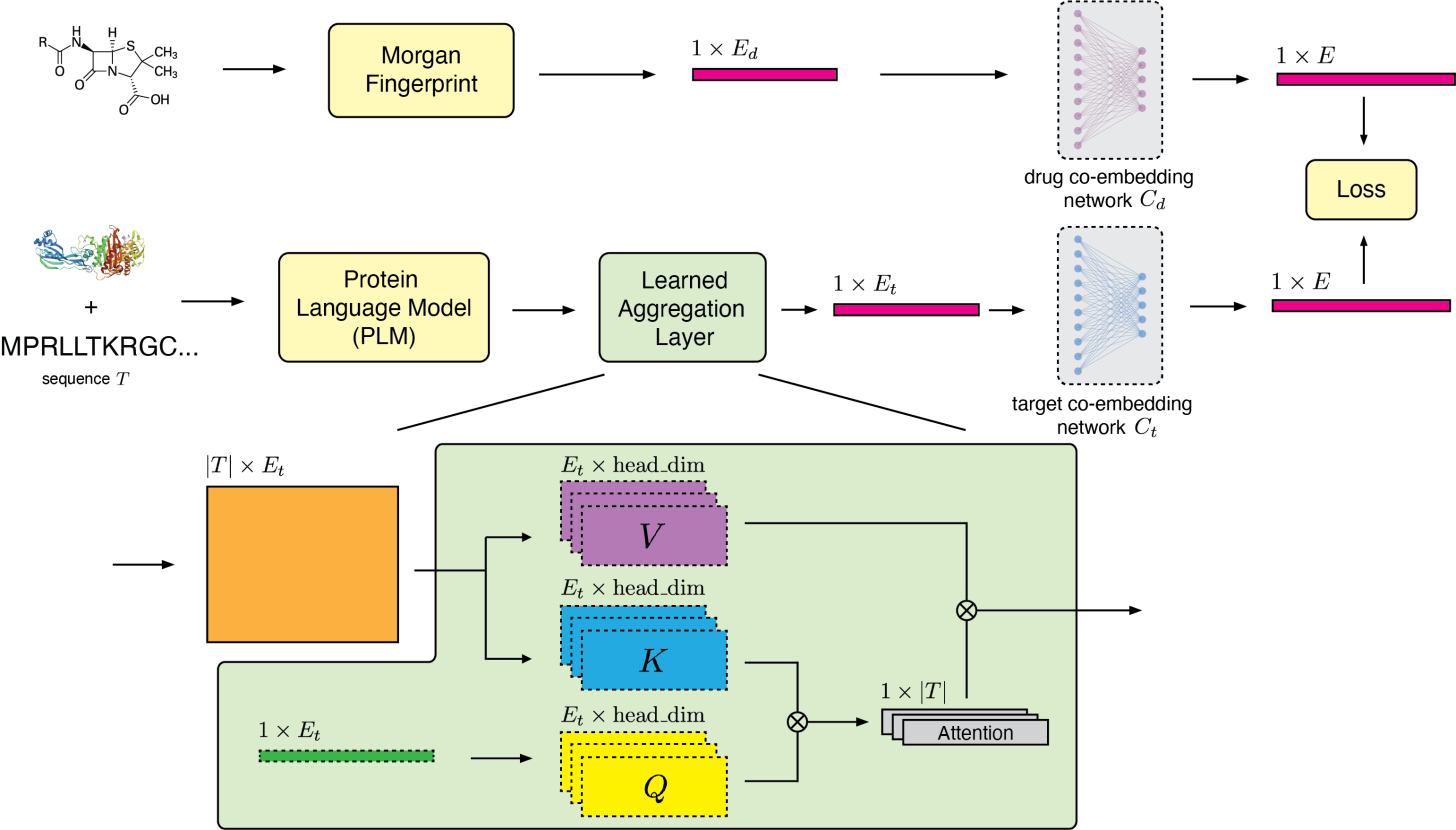
SPRINT learns protein representations via a multi-head attention pooling scheme. Then, SPRINT learns a shared co-embedding space between molecules and protein targets via modality-specific neural networks $C_{d}$ and $C_{t}$. The model is trained end-to-end via a binary cross entropy loss on binding and non-binding drug-target pairs, where the probability of interaction is computed as a sigmoid function of the cosine distance between the drug and target embeddings. The learnable parameters of the network are depicted with dashed borders.

For the drug encoder f, we use the Morgan fingerprint featurizer available in RDKit with bit length 2048 and radius 2 [5, 38]. For the target encoder g, we choose the structure-aware transformer model SaProt [8], a structure-aware protein language model that outputs per-residue embeddings, resulting in a |T|×E featurization for an input sequence T. SaProt optionally takes protein structure as an input to compute FoldSeek tokens for embedding [12]. We utilize AlphaFold2 [39] predicted structures to generate structure tokens when training the DTI model with SaProt. Unlike prior works, we employ multi-head attention pooling to aggregate these per-residue embeddings into a single vector representation of a protein (Fig. 5). This approach has two merits. First, we hypothesize that the model will be able to focus on information-rich residues due to the data-dependent nature of the attention scheme. Second, we can gain insights into the biological relevance of the attention patterns learned by the model by analyzing which residues are prioritized and how they may relate to known mechanisms of drug-target interaction. Further training details and hyperparameters are provided in Appendix C.

### 3.1 Data

We evaluate the performance of the SPRINT DTI prediction architecture on two tasks: interaction prediction and binding affinity prediction. SPRINT models trained for interaction prediction predict 1 if a drug and target pair interact and 0 otherwise. We utilize the same interaction prediction datasets as [2]: DAVIS, BIOSNAP, and BindingDB. DAVIS[40] and BindingDB[41] are both composed of drug-target paired with experimentally annotated dissociation constants, $K_{d}$. We utilize the same thresholds as [2] to set the binary labels: $K_{d}<30$ are labeled as interacting and $K_{d}\geq 30$ are non-interacting. DAVIS consists of only 2086 interactions, BindingDB has 12668, and BIOSNAP has 19238 DTIs. The BIOSNAP[42] data originally consisted of only interacting drug-target pairs, so negative pairs are created by randomly sampling an equal number of drug-target pairs. The Unseen Drugs and Unseen Targets datasets are different splits of BIOSNAP from [43] to elucidate the zero-shot performance of DTI methods. The Unseen Targets dataset is constructed by randomly selecting 20% of the proteins from the full BIOSNAP dataset and assigning all interactions involving these proteins to the test set, while the remaining interactions are used for training. The Unseen Drugs dataset is constructed in an identical manner, but using the drugs rather than the targets.

All dataset splits are the same as [43], 70/10/20 for training, validation, and test, respectively. The training split is artificially subsampled to have an equal number of positive and negative interactions. To evaluate the scaling of the model on more training tokens, we utilize the “MERGED” dataset[16]. This dataset is composed of data from PubChem [17] (98.31%), BindingDB [18] (1.17%), and ChEMBL [19] (0.52%). We use the activity label of the dataset for our interaction label providing 929,656 positive pairs and 83,703,190 negative pairs. We split the data into train, validation, and test splits such that targets in train and validation have at most 90% sequence homology, and targets in train and test have at most 70% sequence homology (splits are further detailed in Appendix C).

We use the Therapeutic Data Commons (TDC)[29] BindingDB_Patent dataset for binding affinity prediction. Each drug-target interaction is labeled with an experimentally determined $IC_{50}$. The BindingDB_Patent dataset splits train and test with a temporal split on the patent date, everything before 2019 is included in the train and validation split, while everything 2019 and after is in the test set. Temporal splits are still prone to data leakage as new drugs are commonly developed for established protein targets as well as drugs being reused for new targets [44]. The TDC hosts a leaderboard for this dataset to continuously evaluate new methods on the same training and test splits. We reserve a random 20% of the training set as a validation set.

## 4 Discussion

Vector-based screens are extremely fast, enabling DTI prediction in regimes that would be impossible with structure-based approaches. We propose SPRINT, which improves on prior work using multi-head attention pooling that scales favorably as we increase the number of DTI training tokens. We show that SPRINT sets a new SOTA for DTI classification, virtual screening, and matches the performance of the top method on the TDC BindingDB_Patent leaderboard (Otter-Knowledge-Ensemble[30]) for binding affinity prediction, without the use of explicit knowledge graphs of protein-ligand interactions. We compare SPRINT using the ESM2 embeddings to their ensemble model using refined protein and ligand representations starting from the same representations as SPRINT. The SPRINT model achieves better binding affinity prediction than their four model ensemble which highlights the value of the attention pooling for the protein and the co-embedding space learned by the model. We demonstrate that SPRINT can perform virtual screening at pan-species proteome scales, e.g., for antimicrobial activities (Fig. A1). Lastly, we find that predicting DTIs via co-embedding is an effective pre-training strategy that enhances simple molecular property prediction (Table B1).

We also show that structure-aware PLMs like SaProt can confer performance gains in virtual screening. Interestingly, we find that the SPRINT models that perform best on the LIT-PCBA virtual screening benchmark, with increased negative sampling, have the least interpretable attention maps. We hypothesize that equal weighting of positive and negative drug-target pairs helps the model learn about residues that interact while increasing the amount of negatives dilutes the information of the positive examples. We compare the virtual screening performance of SPRINT on the second CACHE challenge to the second place computational method which utilizes DeepDocking. Notably, methods using DeepDocking have consistently placed in the top two for the three CACHE challenges in which final results are public, demonstrating its performance for finding hits from large libraries. SPRINT enables direct querying of the entire ENAMINE database, foregoing the inital random sample which can significantly bias the subsequent rounds of DeepDocking. After performing only 1/6th of the docking, SPRINT finds almost three times the number of high-scoring molecular scaffolds compared to gnina DeepDocking. SPRINT’s top molecules can be a useful starting point for DeepDocking to ensure that chemical space is more effectively explored.

We envision the SPRINT framework and training task as a useful benchmarking tool for protein and molecule encoders. Future work will evaluate other structure-aware PLMs such as MULAN or S-PLM [45, 46], and other pre-trained molecule encoders like UniMol [47, 48], in the SPRINT framework for DTI prediction. Additional work to interpret the aggregated protein features through methods like InterPLM[49] could provide insight into the virtual screening predictions. We anticipate this technology will democratize virtual screening by enabling the discovery of lead molecules with a fraction of the compute cost required by comparable, structure-based, virtual screening methods.

## Supplementary Material

Supplement 1

## Figures and Tables

**Table 1:** AUPR on test sets for DTI prediction with co-embedding models across benchmarks (mean ± std). Train, validation, and test splits for BIOSNAP, Unseen Drugs, Unseen Targets, BindingDB, and DAVIS are taken from [2]. The MERGED dataset is split by homology (see Appendix C for more details).

Model	ConPLex	ConPLex-attn*	SPRINT-sm (10M)	SPRINT (16M)

Backbone	ProtBert	ProtBert	SaProt	SaProt
Pooling	Avg	Attn	Attn	Attn

BIOSNAP	0.883±0.004	0.904±0.005	**0.936**±0.001	0.858±0.001
Unseen Drugs	0.874±0.002	0.905±0.002	**0.906**±0.001	0.851±0.002
Unseen Targets	0.842±0.006	0.844±0.011	**0.849**±0.006	0.793±0.007
DAVIS	0.457±0.037	0.493±0.014	**0.507**±0.005	0.446±0.003
BindingDB	0.616±0.009	0.672±0.003	**0.718**±0.0004	0.588±0.0006
MERGED	0.414±0.004*	0.448±0.018	0.481±0.004	**0.526**±0.002

* indicates that we did not do contrastive training on DUD-E with the ConPLex model.

**Table 2:** Virtual Screening results on LIT-PCBA. SPRINT-ProtBert replaces SaProt with the ProtBert model, and SPRINT-Average replaces learned aggregation with average pooling and additional MLP layers. Parameter counts are shown in parentheses.

	AUROC (%)	BEDROC (%)	EF		
			0.5%	1%	5%

Surflex [20]	51.47	-	-	2.50	-
Glide-SP [21]	53.15	4.00	3.17	3.41	2.01
Planet [22]	57.31	-	4.64	3.87	2.43
Gnina [23]	60.93	5.40	-	4.63	-
DeepDTA [24]	56.27	2.53	-	1.47	-
BigBind [25]	60.80	-	-	3.82	-
DrugCLIP [9]	57.17	6.23	8.56	5.51	2.27

SPRINT-Average (15.7M)	67.49	7.80	7.23	6.26	3.71
SPRINT-ProtBert (15.9M)	**73.4**	11.9	11.68	10.19	5.27
SPRINT (16M)	**73.4**	**12.3**	**15.90**	**10.78**	**5.29**

**Table 3:** Pearson’s Correlation Coefficient on the TDC BindingDB_Patent Leaderboard (mean ± std).

Model	Pearson’s Correlation Coefficient

Otter-Knowledge-Ensemble[30]	0.588 ± 0.002

SPRINT-ProtBert	0.593 ± 0.005
SPRINT-ESM2	0.582 ± 0.012
SPRINT	0.588 ± 0.011

**Table 4:** Statistics of DTI datasets

Dataset	unique drugs	unique targets	positive interactions	negative interactions

DAVIS	68	379	1506	9597
BIOSNAP	4510	2181	13836	13647
BindingDB	10665	1413	9166	23435
MERGED	3529623	11958	929656	83703190

BindingDB_Patent	140746	477	N/A	N/A

## Data Availability

DAVIS, BIOSNAP, BindingDB, and MERGED DTI classification datasets are available through our github: https://github.com/abhinadduri/panspecies-dti. The BindingDB DTI DG dataset for the TDC Leaderboard is available through the Therapeutics Data Commons: https://tdcommons.ai/. The Lit-PCBA evaluation benchmark is available through the original authors https://drugdesign.unistra.fr/LIT-PCBA/. The filtered Enamine REAL dataset used for the second CACHE challenge is too large for upload, but can be obtained from the authors upon reasonable request.
