# Supplementary material for: Scaling Structure Aware Virtual Screening to Billions of Molecules with SPRINT
Source: ArXiv. 2025 Jan 20:arXiv:2411.15418v2. Preprint. [Version 2] (PMC11838698)
Supplement: Supplement 1 [file NIHPP2411.15418v2-supplement-1.pdf]

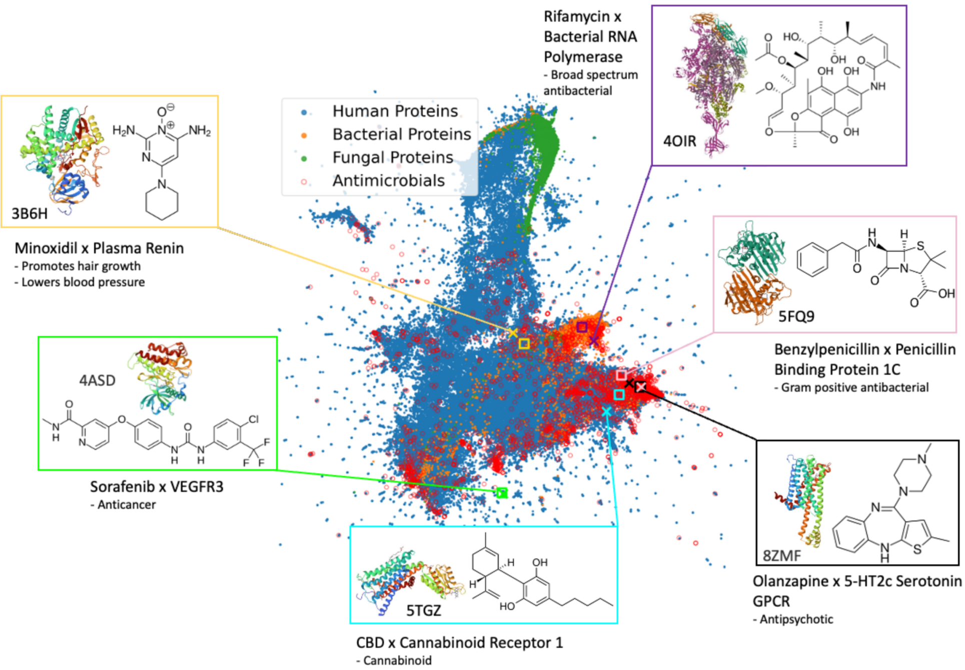

Fig. A1 UMAP visualization of the binding co-embedding space of drug-like small molecules and their protein targets across bacterial, fungal, and human proteomes. We see that antimicrobial compounds co-localize with regions of the shared latent space that contain human, bacterial, and fungal proteomes.

Appendix A SPRINT recovers known mechanisms of action

Our pan-species protein dataset is comprised of all predicted protein sequences in reference genomes from NCBI within the taxons bacteria, fungus, and human. Each taxon contained 3,379,854, 775,477, and 136,194 protein sequences respectively. We gathered a list of 3,112 natural products [37], as they are known to have a high prior likelihood for antimicrobial activity [50] and are out-of-distribution relative to our MERGED training dataset. We then co-visualized several antimicrobials and drugs with their known protein targets across microbial proteomes (Fig. A1), recovering several known mechanisms of action. The dataset is available to query at https://bit.ly/colab-screen.

Appendix B SPRINT co-embeddings improve property prediction

Current antimicrobial and toxicity screening approaches are often formulated as molecular property predictors, framing antimicrobial activity and toxicity to humans as inherent properties of drug molecules [36, 51–55]. Our results demonstrate that

Table B1 F1 scores for MLP classification models applied to molecule embedding strategies (mean ± std). Models trained on Morgan fingerprints used a larger hidden size to match the size of models trained on fingerprints concatenated with embeddings.

Featurization	Antibacterial Task	Toxicity Task
Morgan Fingerprint	0.740 ± 0.027	0.720 ± 0.008
SPRINT-sm Embedding	0.687 ± 0.012	0.656 ± 0.015
Morgan Fingerprint + SPRINT-sm Embedding	**0.749** ± 0.016	**0.735** ± 0.006
SPRINT Embedding	0.614 ± 0.027	0.631 ± 0.023
Morgan Fingerprint + SPRINT Embedding	0.722 ± 0.027	0.701 ± 0.018

augmenting Morgan fingerprints with SPRINT-sm ligand embeddings consistently outperformed an equivalently sized neural network using Morgan fingerprints alone, when evaluated on both an antibacterial activity dataset [37] and a toxicity dataset [36] (Table B1). We hypothesize that vectorizing the DTI space allows property prediction methods to leverage information about target neighborhoods around a drug, enhancing performance and offering mechanistic explanations for these properties based on likely binding partners. The embeddings from the deeper SPRINT model consistently performed worse than those from the shallow SPRINT-sm model, suggesting that shallow transformations of the Morgan fingerprint work best in this setting. The standalone SPRINT embeddings achieved substantially lower performance than their concatenated counterparts, indicating that SPRINT embeddings may capture complementary molecular features to traditional fingerprints.

Appendix C Training details

DTI models are trained using the same train/val/test splits as [2] for the DAVIS, BindingDB, and BIOSNAP datasets. All structure tokens for SaProt were computed on AlphaFold2 [39] generated structures. Structures were downloaded from the AlphaFold Protein Structure Database if they existed. When no precomputed structure was available, ColabFold [56] was run with 2 random seeds to generate 10 energy minimized structures, and the minimized structure with the highest pLDDT was used. Structure tokens were generated with Foldseek[12], masking the structure token if the residue pLDDT was less than 70.

Specifically, the loss $\mathcal{L}$ is written as

$$\mathcal{L} = \frac{1}{N}\sum_{i}^{N}\left[Y^i \log(\tilde{Y}^i) + (1 - Y^i)\log(1 - \tilde{Y}^i)\right] \tag{C1}$$

$$\tilde{Y}^i = P(Y^i = 1 | Z_d^i, Z_t^i) = \sigma\left(\alpha \frac{Z_t^i}{\|Z_t^i\|} \cdot \frac{Z_d^i}{\|Z_d^i\|}\right) \tag{C2}$$

Table C2 LIT-PCBA evaluation ablation of negative sampling. '1:1' indicates equal sampling of positive and negative examples during training and '3:1' indicates the preferred model training with 3 negatives sampled for every positive example.

Model	AUROC	BEDROC ($\alpha = 0.85$)	EF (0.5%)	EF (1%)	EF (5%)
SPRINT-ProtBert 1:1	71.53	7.78	6.81	5.87	3.86
SPRINT 1:1	72.71	10.16	10.31	8.86	4.73
SPRINT-ProtBert 3:1	**73.4**	11.9	11.68	10.19	5.27
SPRINT 3:1	**73.4**	**12.3**	**15.90**	**10.78**	**5.29**

where the protein, T^i, and drug, D^i, have been mapped to the SPRINT co-embedding space as Z_t^i and Z_d^i, respectively. $Y_i \in \{0, 1\}$ is a ground-truth label with value 1 if T^i and D^i are binders or 0 if they are non-binders. The pre-sigmoid scalar value, α, is used to expand the range of cosine-similarity to the domain of the sigmoid. We set α to 5. Models are trained for binary classification using standard, supervised cross-entropy loss.

Binding affinity regression models use the same architecture to learn the co-embedding space, but the final, non-learned components are removed. The sigmoid and pre-sigmoid scalar value are removed and the cosine similarity is replaced with a dot-product (Equation 2). This allows the magnitude of drug and target embeddings to impact the prediction as well as increasing the range to all real numbers. Regression models are trained with mean squared error loss.

ConPLex models are trained with the hyperparameters used in the original paper [2]. Our Attention Pooling models are trained with a learning rate of 1×10^{-5} and a dropout value of 0.05 for 250 epochs, keeping all other hyperparameters the same as ConPLex training. We set weight decay to 0.01 for the binding affinity regression model following hyperparameter tuning on the validation set. The model checkpoint with the highest validation AUPR for classification models and MSE for regression models during training is evaluated on the test set (Table 1 and 3).

To enable efficient training on the MERGED dataset, we featurize the unique proteins and molecules before training, storing their representations in memory-mapped files for quick retrieval using the Lightning Memory-Mapped Database Manager (LMDB) library. To address data imbalance in the binding data, for each epoch, we train using all of the drug-target binding pairs, and subsample an equivalent number of non-binding pairs without replacement. We observed that models trained with more negatives than positives (at a 3:1 ratio), achieved better virtual screening performance, but had less interpretable attention patterns (Tables 1, C2). Models are trained for 20 epochs. All other hyperparameters were kept the same.

The MERGED dataset splits were determined by clustering protein sequences using MMSeqs2 [28] at 80% coverage threshold and 70% sequence identity, meaning two sequences appear in the same cluster if at least 80% of residues are aligned with at least 70% identity. Clusters were then assigned to splits by size, with smaller clusters preferentially assigned to test and validation sets until each contained approximately 10% of the total number of unique proteins. The remaining sequences were assigned to training. Drug-target interactions were then partitioned according to their protein

assignments. The final training, validation, and test sets contained 79.5%, 10.3%, and 10.2% of total interactions, respectively.

Appendix D High scoring molecules screened with SPRINT for NSP13 Helicase

We show the 2D depiction of the highest scoring molecules, according to GNINA's CNN VS, from SPRINT's screen of Enamine REAL in Figure D2. We see that the molecules represent diverse scaffolds.

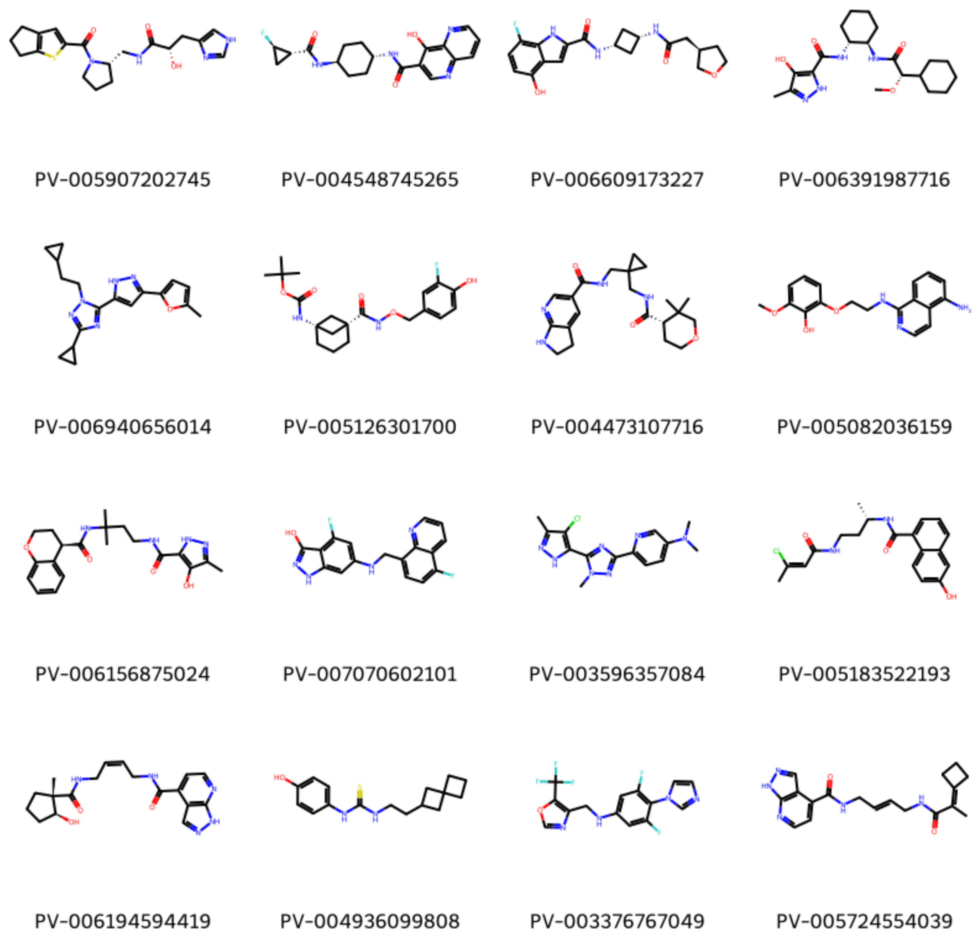

Fig. D2 2D depiction of the high-scoring (CNN VS > 6) molecules found by SPRINT for NSP13 Helicase.

Appendix E Investigating the learned aggregation layer

We investigate the attention pattern of our learned aggregation layer and compute the relative weighting of binding and non-binding residues. For this analysis, we use the intersection of the PDBbind refined v.2019 [57] dataset and the dataset created by [58]. The intersection of these datasets provides 109 single-chain, high-quality protein-ligand binding structures with annotated binding sites. Following the same protocol as [58], we determine binding residues based on a maximum heavy-atom distance of 5 Å between the residue and the ligand.

We first analyzed the attention patterns of each head in the learned aggregation layer to determine if any of the heads were selective for binding residues. We calculate the attention scores for each residue in the protein and compute the mean attention value for the binding residues and the non-binding residues. Figure 2 shows the average weight of the binding residues and non-binding residues across the dataset for the ProtBert and SaProt models trained with equal positive and negative sampling. We find the models trained with equal positive and negative sampling have more interpretable attention maps despite a decreased performance on the LIT-PCBA benchmark compared to models trained with more negative samplings. We compare the attention of all the models in Figure E3, visualizing three otherwise identical models trained with different initial random seeds. Interestingly, the PLM with the worst performance on the LIT-PCBA benchmark, ProtBert, shows the most attention to binding site residues relative to its attention to non-binding site residues. The structure-aware PLM, SaProt, has two seeds that attend to binding residues more than non-binding residues across most of the attention heads and one seed that pays very little attention to the binding residues. The SaProt seed that has the least attention for binding residues as compared to non-binding residues performs the best on the LIT-PCBA benchmark. Across all PLMs, there is a large variance in the attention to binding residues as the models initial random seed is changed.

We visualize the learned aggregation layers attention heads on the protein-ligand structures for both ProtBert and SaProt models in Figure 3 with additional visualizations provided in Appendix F and on our github.

Appendix F Structural visualizations

We visualize the attention patterns of the attention pooling layer on the protein-ligand bound structure of several PDB IDs. We compare the attention patterns of SPRINT models with ProtBert and SaProt trained on the MERGED dataset. We see across these diverse proteins and ligands that on average, the SaProt model attends to residues closer to the ligand, while the ProtBert models often attend to residues far from the binding site.

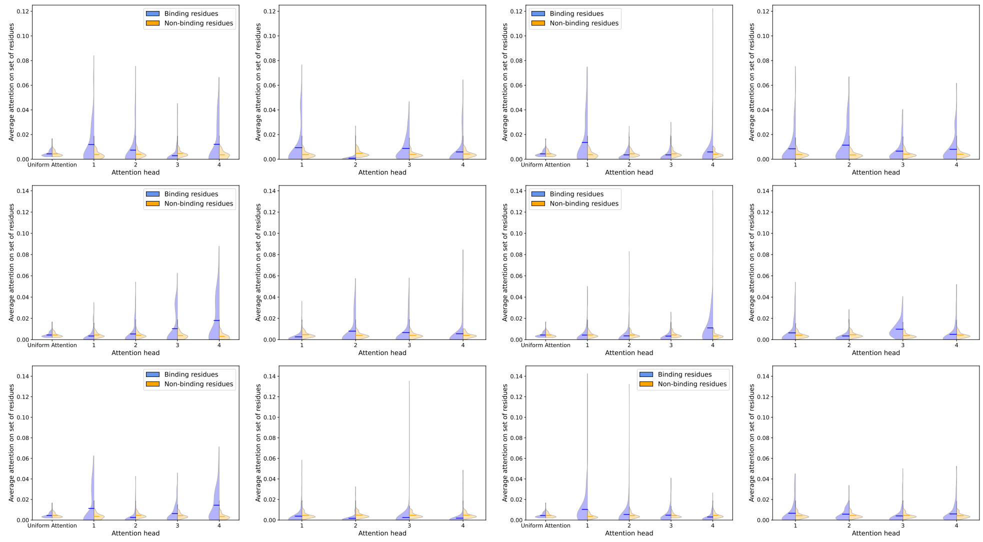

(a) ProtBert (b) SaProt (c) ProtBert-1to1 (d) SaProt-1to1

Fig. E3 Comparing the average attention weight of binding and non-binding residues on our set of 109 single-chain protein-ligand binding structures after training on the MERGED Dataset. The horizontal line indicates the average across the proteins. Each row is a different random seed and each column is a different PLM or different training regime, where '1to1' indicates that a 1:1 positive to negative sampling ratio was used during training.

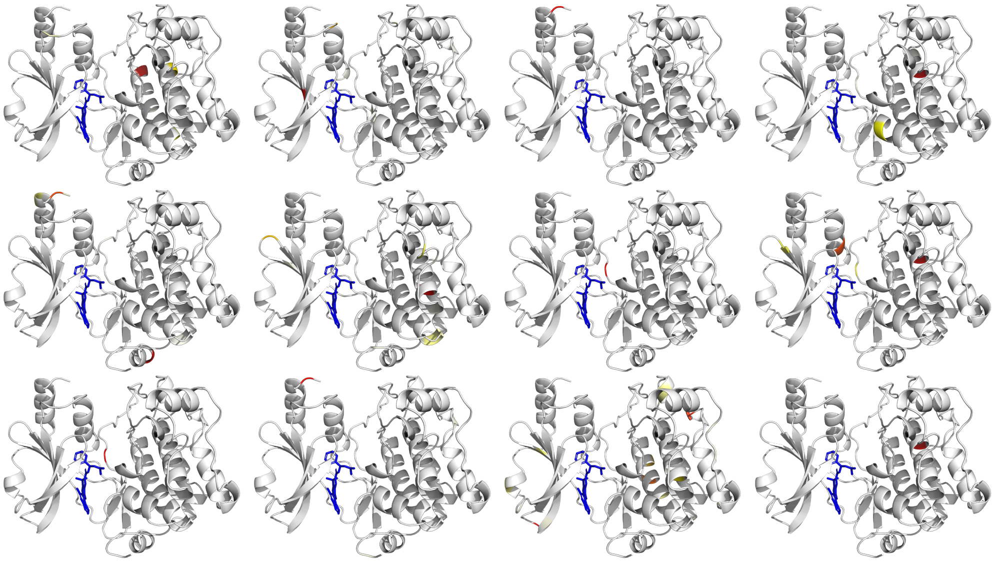

Head 1 Head 2 Head 3 Head 4

Fig. F4 Analyzing the attention of the ProtBert model on PDB ID 2X4Z. Each row is the ProtBert model trained with different seed. Each column is a different attention head. Gradient from white to red indicates the attention weight, where white is no attention and red is max attention for that head. The ligand is shown in blue.

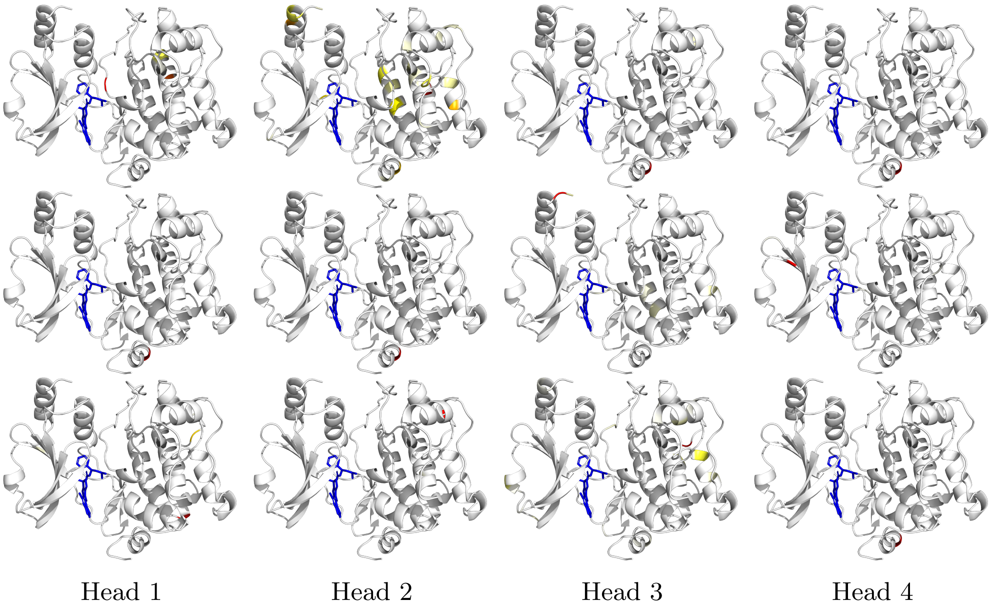

Fig. F5 Analyzing the attention of the ProtBert-1to1 model (trained with 1:1 positive to negative ratio) on PDB ID 2X4Z. Each row is the ProtBert model trained with different seed. Each column is a different attention head. Gradient from white to red indicates the attention weight, where white is no attention and red is max attention for that head. The ligand is shown in blue.

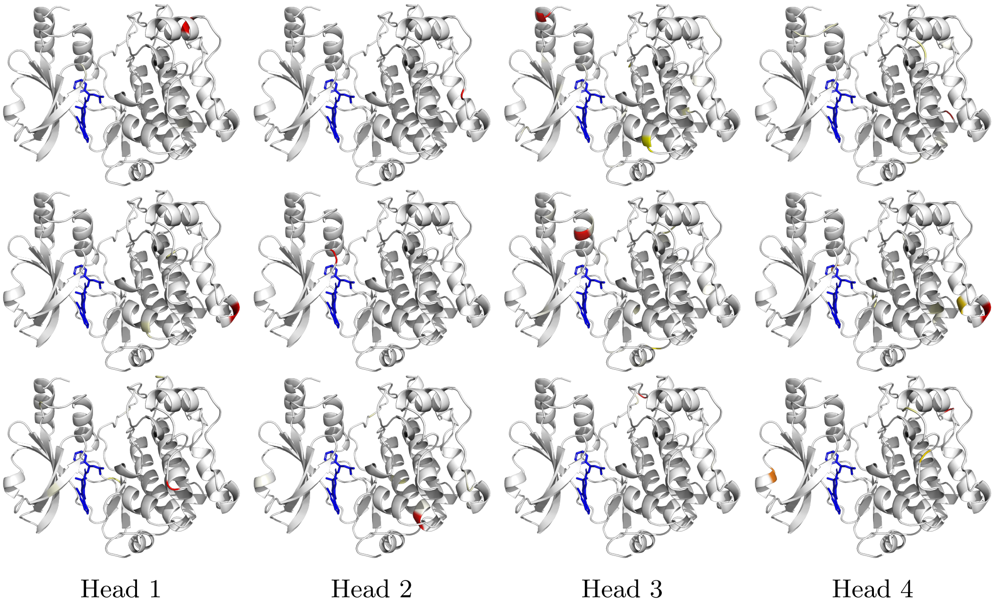

Fig. F6 Analyzing the attention of the SaProt model on PDB ID 2X4Z. Each row is the SaProt model trained with different seed. Each column is a different attention head. Gradient from white to red indicates the attention weight, where white is no attention and red is max attention for that head. The ligand is shown in blue.

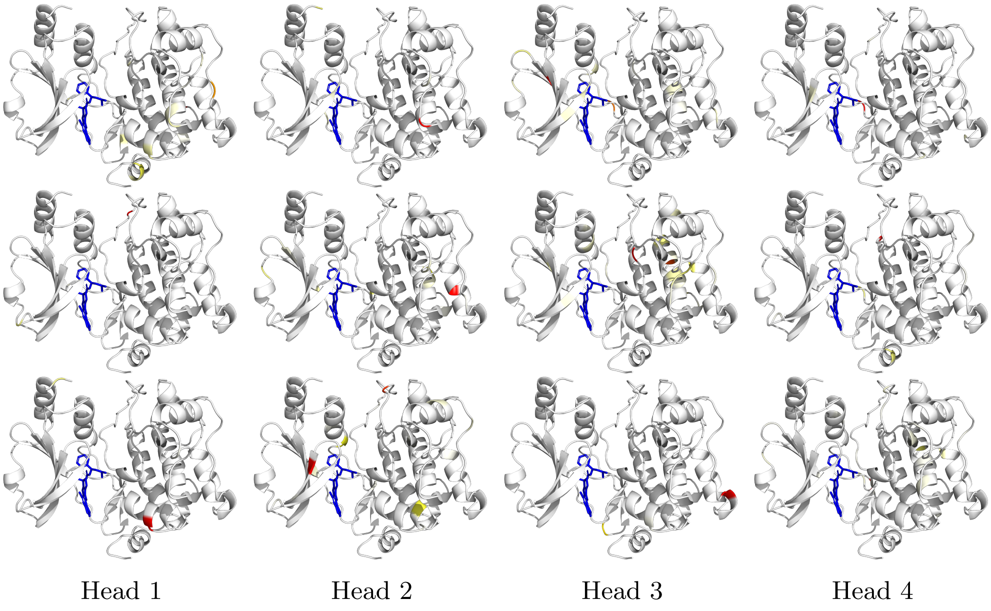

Fig. F7 Analyzing the attention of the SaProt-1to1 model (trained with 1:1 positive to negative ratio) on PDB ID 2X4Z. Each row is the SaProt model trained with different seed. Each column is a different attention head. Gradient from white to red indicates the attention weight, where white is no attention and red is max attention for that head. The ligand is shown in blue.
